# Antibacterial Efficacy of Artemisia Extract and White Vinegar as Disinfectants for Acrylic Prostheses Fabricated Using Conventional and CAD/CAM Techniques: An In Vitro Study

**DOI:** 10.1155/ijod/6892571

**Published:** 2026-08-12

**Authors:** Mariette Droubi, Joul Kassis, Alaa salloum, Jalal Fandi, Samar Alsalameh

**Affiliations:** ^1^ Department of Removable Prosthodontics, Faculty of Dentistry, Damascus University, Damascus, Syria, damascusuniversity.edu.sy; ^2^ Department of Oral and Maxillofacial Surgery, Faculty of Dentistry, Damascus University, Damascus, Syria, damascusuniversity.edu.sy; ^3^ Department of Regional Development of Natural Resources and Environmental Protection, The Higher Institute of Regional Planning, Damascus University, Damascus, Syria, damascusuniversity.edu.sy; ^4^ Department of Basic Science, Faculty of Dentistry, Damascus University, Damascus, Syria, damascusuniversity.edu.sy

**Keywords:** *Artemisia herba-alba*, CAD/CAM acrylic resin, denture-related stomatitis, heat-polymerized acrylic resin, *Staphylococcus aureus*

## Abstract

**Objective:**

To evaluate the antibacterial efficacy of *Artemisia herba-alba* extract and white vinegar as natural disinfectants against *Staphylococcus aureus* on computer‐aided design and computer‐aided manufacturing (CAD/CAM)‐milled and heat‐polymerized denture base resins.

**Materials and Methods:**

Sixty acrylic resin blocks were prepared and divided into two main groups (*n* = 30 each): CAD/CAM‐milled and heat‐polymerized acrylic resin. The blocks were contaminated with *Staphylococcus aureus* isolated from a patient with denture‐related stomatitis and immersed for 60 min in one of three solutions: *Artemisia herba-alba* extract, white vinegar, or distilled water (negative control). Antibacterial efficacy was assessed by quantifying colony forming units (CFUs) using the viable count method.

**Results:**

A Kruskal–Wallis test revealed that both *Artemisia herba-alba* extract and white vinegar significantly lowered CFU counts relative to the control (*p* < 0.05) across both material groups. Notably, the *Artemisia herba-alba* extract exhibited superior antibacterial efficacy compared to white vinegar, yielding log reductions of 4.11 and 3.42 in heat‐polymerized and CAD/CAM acrylic specimens, respectively. In contrast, white vinegar produced more modest log reductions of 1.81 and 1.20, respectively. Under the studied conditions, heat‐polymerized acrylic consistently demonstrated slightly higher bacterial reduction than CAD/CAM acrylic.

**Conclusion:**

Both *Artemisia herba-alba* extract and white vinegar demonstrated antibacterial activity against *Staphylococcus aureus* on removable denture base materials, with the extract demonstrating superior potency. While heat‐polymerized acrylic showed slightly higher bacterial reduction than CAD/CAM acrylic, both material types responded positively to the tested disinfecting agents.

**Significance:**

This study demonstrates that *Artemisia herba-alba* extract and white vinegar are effective natural disinfectants against *Staphylococcus aureus* associated with denture materials. It supports their use as low‐cost natural alternatives for managing denture stomatitis and improving denture hygiene. Alternatively, it highlights their potential as low‐cost natural alternatives for improving denture hygiene and warrants further investigation regarding clinical applicability and long‐term safety.

## 1. Introduction

Tooth loss negatively affects an individual’s quality of life as it leads to functional difficulties in mastication and speech, in addition to esthetic and psychological consequences [1].

Although various treatment options are available to replace missing teeth, including fixed and removable prostheses, health‐related and economic limitations, particularly among elderly patients, often restrict the use of dental implants or fixed restorations. Consequently, removable dental prostheses remain the most appropriate treatment option due to their lower cost and suitability for a wide range of systemic conditions.

Polymethyl methacrylate (PMMA) remains the material of choice for fabricating complete and partial denture bases due to its straightforward processing and satisfactory mechanical properties. However, PMMA exhibits certain surface‐related drawbacks. Recent advancements in computer‐aided design and computer‐aided manufacturing (CAD/CAM) technologies have contributed to improving denture fabrication and reducing some of the limitations associated with conventional methods [2, 3].

Conventional acrylic denture bases often have a rough, porous fitting surface, which promotes microbial attachment and biofilm formation. This provides an ideal niche for opportunistic bacteria like *Staphylococcus aureus* (*S. aureus*) to adhere and multiply. Indeed, studies report that denture wearers typically harbor higher *S. aureus* colonization than individuals without dentures [4, 5]. Unremoved biofilms on dentures can trigger denture stomatitis, a common inflammatory condition of the oral mucosa [6].

To maintain denture hygiene and prevent denture stomatitis, mechanical and/or chemical cleaning methods are commonly recommended. However, mechanical cleaning can be challenging for elderly patients due to reduced manual dexterity, making chemical disinfectants a practical alternative. Sodium hypochlorite at a concentration of 0.5% is widely used because of its effectiveness in reducing microbial plaque without significantly affecting the physical and mechanical properties of denture base materials. Nevertheless, prolonged or daily use of chemical disinfectants may adversely affect denture bases, increasing surface roughness and promoting microbial adhesion [7, 8].

As a result, there is growing interest in exploring natural, cost‐effective, and easily accessible alternatives to chemical disinfectants that may exert fewer adverse effects on the physical and mechanical properties of denture base materials. Among these alternatives are medicinal plant extracts, such as *Artemisia herba-alba* and white vinegar. *Artemisia herba-alba* (desert wormwood) is an aromatic herb long used in traditional medicine. Its essential oil (EO) contains bioactive terpenoids, notably α‐ and β‐thujone, camphor, and cineole, which are believed to underlie its broad‐spectrum antimicrobial effects [9, 10]. White vinegar (5%–10% acetic acid) is a simple, natural antimicrobial that can inactivate a range of microbes. Studies have shown that acetic acid solutions can significantly reduce *S. aureus* counts on treated surfaces [11, 12].

This study aims to evaluate the antibacterial effects of *Artemisia herba-alba* extract and white vinegar against *S. aureus* associated with denture stomatitis and to compare their effectiveness according to the denture base material, the conventional heat‐polymerized acrylic resin, and the prepolymerized CAD/CAM acrylic resin.

## 2. Materials and Methods

**Figure 1:**
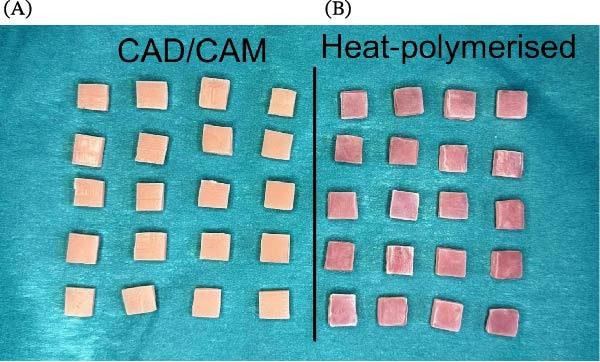
Part of the study sample showing CAD/CAM‐milled acrylic resin (PMMA) (A) and conventional heat‐polymerized resin (BMS) (B).

A total of 60 acrylic resin specimens were prepared for this in vitro study. Of these, 30 specimens were fabricated from prepolymerized CAD/CAM‐milled acrylic resin (PMMA), and 30 specimens were made from conventional heat‐polymerized resin (BMS). All specimens were standardized in the form of rectangular blocks with dimensions of 10 mm × 10 mm × 3 mm (length × width × thickness) (Figure 1). Prior to experimentation, the specimens were sterilized using moist heat sterilization.

The specimens were initially divided into two main groups according to the type of acrylic resin:

Group I (heat‐polymerized): conventional heat‐polymerized acrylic resin specimens (BMS) (*n* = 30).

Group II (CAD/CAM): prepolymerized (CAD/CAM‐milled acrylic resin) specimens (PMMA) (*n* = 30).

Each main group was further divided into three subgroups according to the disinfectant solution used, with 10 specimens assigned to each subgroup (*n* = 10):

Subgroup A: *Artemisia herba-alba* extract.

Subgroup B: White vinegar (commercial vinegar containing 5% acetic acid).

Subgroup C: Control group (distilled water).

Study groups are as follows: Group 1: heat‐polymerized—A, Group 2: heat‐polymerized—B, Group 3: heat‐polymerized—C, Group 4: CAD/CAM—A, Group 5: CAD/CAM—B, and Group 6: CAD/CAM—C.

### 2.1. Pilot Study and Control Validation

Prior to the main experiment, a pilot study confirmed that the dimethyl sulfoxide (DMSO) solvent possessed no independent antibacterial activity against *S. aureus*. Consequently, a separate DMSO control was excluded from the primary study, and distilled water was utilized as the sole negative control. This validated that any observed antimicrobial effects were strictly attributable to the *Artemisia herba-alba* extract.

### 2.2. Methodology and Results

#### 2.2.1. Sample Preparation

Eight heat‐polymerized acrylic resin specimens were divided into two groups (*n* = 4).

#### 2.2.2. Procedure

One group was submerged in distilled water (negative control), while the other was immersed in DMSO solution for 1 h.

This in vitro study was conducted and reported in accordance with the checklist for reporting in vitro studies (CRIS) guidelines. The completed CRIS checklist is provided (Supporting Information 1: File 1).

#### 2.2.3. Testing

All samples were then subjected to standard microbiological analyses.

The data showed no significant difference in microbial growth between the two groups. This confirms that the concentration of DMSO used lacks antibacterial efficacy, verifying that the emulsion’s performance is entirely attributable to *Artemisia herba-alba* EO.

### 2.3. Specimen Preparation

#### 2.3.1. Heat‐Polymerized Acrylic Resin

They were fabricated by the conventional compression molding technique using heat‐polymerized acrylic resin (BMS Dental, Italy). Wax patterns of standardized dimensions were invested in metal flasks and polymerized according to the manufacturer’s instructions for the resin used. Following polymerization, the flasks were allowed to cool in a curing water bath for 24 h before deflasking. The specimens were finished using carborundum burs and polished on one surface only using waterproof abrasive papers (TOA, Thailand), while the opposite surface was left unpolished to simulate the intaglio surface of removable dentures in accordance with ISO 20795‐1:2013 guidelines.

#### 2.3.2. CAD/CAM‐Milled Acrylic Resin

They were digitally designed in the form of rectangular blocks with dimensions of 10 mm in length and width and 3 mm in thickness using Exocad software (Galway 3.0) and milled from prepolymerized PMMA discs (BiLKiM CO. Ltd, Turkey) using a CAD/CAM milling unit (Roland, DWX‐68D).

### 2.4. Extraction and Preparation of *Artemisia herba-alba*

**Figure 2:**
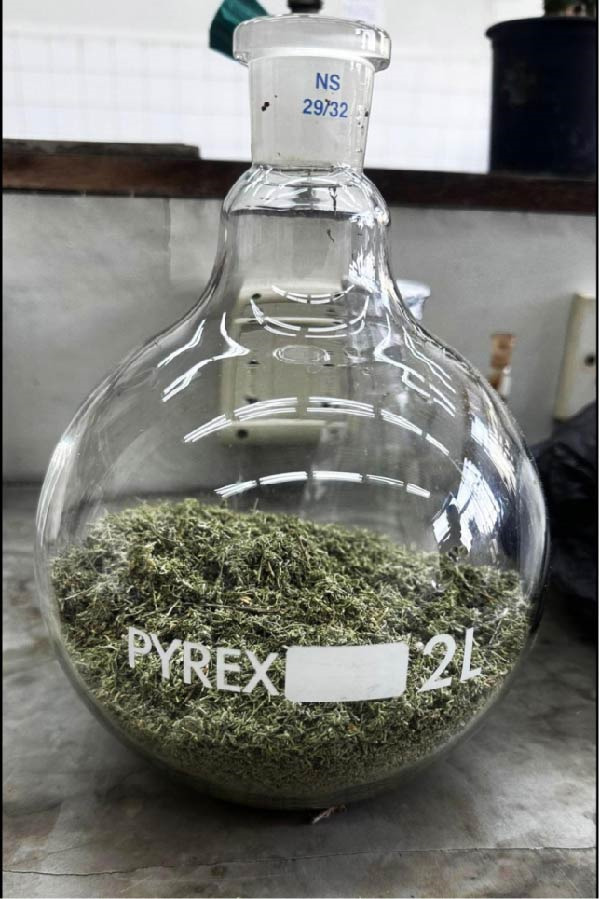
Ground *Artemisia herba-alba* plant.

The EO of *Artemisia herba-alba* was extracted at the Department of Pharmacognosy, Faculty of Pharmacy. The plant material was collected from a village named Maaloula. The geographic origin of the plant material was recorded to improve reproducibility as variations in environmental conditions may influence the chemical composition and biological activity of EOs. The aerial parts of the plant were air‐dried in the dark at room temperature for 20 days and subsequently finely ground (Figure 2). A total of 125 g of the dried plant material was used in two stages. The mass was accurately determined using an electronic balance with a precision of 0.0001 g (RAWAG Balances and Scales, Poland). Then, hydrodistillation was carried out for 3 h using a Clevenger‐type apparatus (DWK Life Sciences, Schott/Duran, Germany). The extracted oil was stored in airtight amber vials at 4°C.

Based on the preliminary pilot study, a final concentration of 0.5% (v/v) was selected for the main experiment as both 0.5% and higher tested concentrations demonstrated effective antibacterial activity. The lower effective concentration was chosen to minimize the amount of EO required while maintaining antimicrobial efficacy. To achieve a final concentration of 0.5% (v/v) [10], 0.5 mL of pure *Artemisia herba-alba* EO was first dispersed in a small amount of DMSO to ensure proper solubility and emulsification. This mixture was then combined with distilled water through titration until reaching a total volume of 100 mL. To address potential solvent interference, during the pilot study, a DMSO‐containing solution prepared at the same ratio used for the Artemisia emulsion was tested and showed no detectable antibacterial activity against *S. aureus*. Therefore, a separate DMSO control group was excluded from the main study, and distilled water was used as the negative control [10].

### 2.5. Isolation of *S. aureus* and Biofilm Formation

*S. aureus* was isolated from oral swabs (Surgeine Healthcare, India) obtained from 10 patients diagnosed with type‐II denture stomatitis at the Department of Removable Prosthodontics, Faculty of Dentistry. The study was conducted in compliance with the World Medical Association Declaration of Helsinki on medical research ethics and approved by the Biomedical Research Ethics Committee (Reference Number DN‐230426‐770). Additionally, written informed consent was obtained from all participants prior to their inclusion in the study.

**Figure 3:**
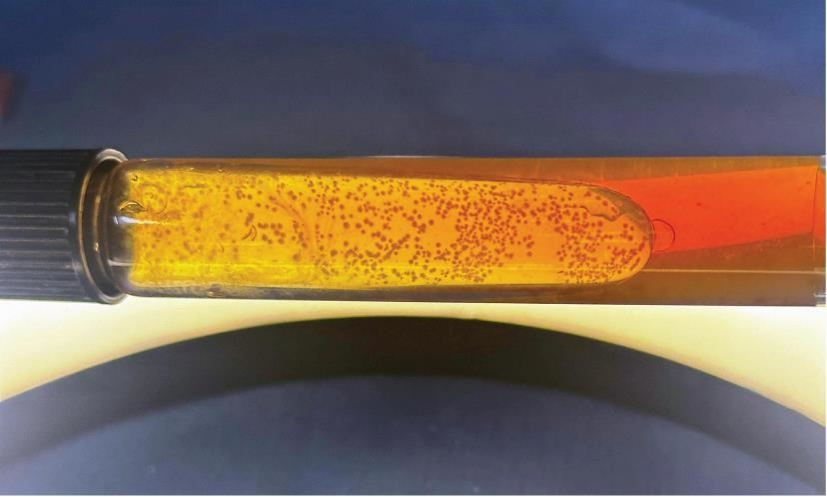
Following the isolation of *S. aureus* on nutrient agar (NA) slants for preservation.

Samples were cultured on Chapman agar (Abtek, UK) and incubated at 37°C for 24 h (Carbolite, Germany). A single confirmed clinical isolate of *S. aureus* was selected and used throughout the experimental procedures. Following confirmation, the isolate was subcultured on nutrient agar slants and stored at 4°C until further use to maintain bacterial viability and ensure consistency throughout the experiments (Figure 3). To confirm the identity of the isolate, presumptive colonies (1–3 mm in diameter) were subjected to a stepwise phenotypic identification protocol. Initial screening was performed using Gram staining, which demonstrated Gram‐positive cocci arranged in grape‐like clusters, consistent with staphylococcal morphology. Catalase testing was subsequently conducted by applying 3% hydrogen peroxide (H_2_O_2_) to a fresh bacterial smear on a glass slide, where immediate effervescence confirmed catalase activity and differentiated staphylococci from catalase‐negative streptococci.

For further phenotypic confirmation, isolates were cultured on mannitol salt agar (Chapman medium), a selective and differential medium for staphylococci. Growth on the high‐salt medium indicated salt tolerance, while mannitol fermentation was evidenced by a pH‐dependent phenol red indicator shift, resulting in a yellow coloration of the medium. This combined selective and differential response supported the presumptive identification of *Staphylococcus aureus*. Confirmed isolates were subsequently subcultured on nutrient agar slants and stored at 4°C until further analysis.

For biofilm formation, acrylic specimens were immersed in sterile nutrient broth (Conda, Spain) inoculated with a standardized suspension of *S. aureus* (adjusted to a 0.5 McFarland turbidity standard, ~1.5/times 108 colony forming unit (CFU)/mL). All acrylic specimens were inoculated using the same bacterial suspension and incubated under identical conditions to ensure a standardized microbial challenge across all experimental groups. The specimens were incubated statically at 37°C for a duration of 48 h to allow for the development of a mature and stable biofilm. After incubation, specimens were carefully rinsed with sterile saline to remove planktonic (nonadherent) bacteria before proceeding with the experimental treatments.

### 2.6. Disinfection Procedure and Microbiological Evaluation

**Figure 4:**
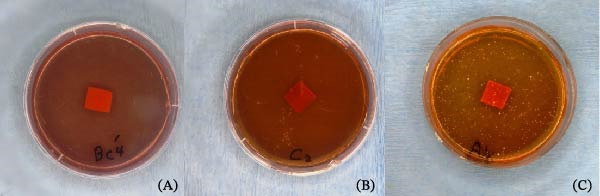
*S. aureus* colonies following treatment with (A) *Artemisia herba-alba*, (B) white vinegar, and (C) distilled water.

Both heat‐polymerized and CAD/CAM‐milled acrylic specimens were divided into three subgroups. Specimens were equally distributed among the three treatment groups (*n* = 10 per subgroup) under standardized experimental conditions to minimize the variability between groups. During the experimental procedure, heat‐polymerized and CAD/CAM acrylic specimens were simultaneously subjected to the same disinfection solutions (*Artemisia herba-alba* emulsion, white vinegar, and distilled water) to ensure comparable exposure conditions and reduce potential bias related to differences in bacterial viability or experimental timing. Specimens were immersed for 1 h in one of the following solutions: *Artemisia herba-alba* emulsion, commercial white vinegar (Al‐Ahlam, Syria), and distilled water (Figure 4A–C).

Following disinfection, each specimen was transferred to a sterile tube containing 1 mL of sterile saline and vortexed for 30 s to detach adherent bacteria. Serial tenfold dilutions were prepared, and 100 μL from each dilution was plated onto Chapman agar and incubated at 37°C for 24 h. Bacterial viability was assessed by counting CFUs using the viable colony count method, which is considered a reliable indicator of antibacterial efficacy against adherent microorganisms [13]. Since a single confirmed clinical isolate of *S. aureus* was used throughout the study, all specimens were inoculated using the same bacterial suspension to ensure a standardized microbial challenge. Each acrylic specimen represented an independent experimental unit, while CFU enumeration was performed using standardized microbiological procedures to ensure measurement reliability and reproducibility.

#### 2.6.1. Sample Size Calculation

A sample size calculation was conducted using 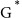
Power software (version 3.1.9.7). The input parameters of effect size (*f* = 0.80, *α* = 0.05, and power = 0.80) for comparing three independent groups using one‐way ANOVA (fixed effects, omnibus), with. The minimum required sample size was 21 specimens (7 per group) with critical *F* = 3.5545571, numerator *df* = 2, and denominator *df* = 18 as output parameters. To improve reliability and compensate for possible experimental variability, 10 specimens were included in each subgroup.

#### 2.6.2. Statistical Analysis

Statistical analyses were performed using SPSS software (Version 27.0; IBM Corp.). The Shapiro–Wilk test was used to assess the data distribution. Since the data did not meet parametric assumptions, comparisons among the three disinfection groups were performed using the Kruskal–Wallis test (Table 1), followed by Dunn’s post hoc test with Bonferroni adjustment for multiple comparisons. Differences between the two acrylic fabrication methods within comparable treatment conditions were evaluated using the Mann–Whitney *U* test. Statistical significance was set at *α* = 0.05 (Supporting Information 2: File 2).

**Table 1:** The results of the data normality test for the studied groups.

Group	Test statistic	Degrees of freedom (*df*)	Significance (sig.)	Distribution type
Thermal—A	0.42	10	<0.001^∗^	Nonnormal
Thermal—B	0.96	10	0.780	Normal
Thermal—C	0.91	10	0.290	Normal
CAD/CAM—A	0.41	10	<0.001^∗^	Nonnormal
CAD/CAM—B	0.99	10	0.990	Normal
CAD/CAM—C	0.89	10	0.170	Normal

^∗^Statistical significance for the Kruskal‐Wallis test across treatments.

#### 2.6.3. Descriptive Statistics

After counting the CFUs for all groups, the raw data were transformed into logarithmic values using the equation log_10_(CFU + 1) to accommodate zero counts prior to the statistical analysis. The data for each group were compiled separately, allowing comparison of bacterial survival among experimental groups. Results were presented as mean ± standard deviation (SD), median with interquartile range (IQR), when appropriate, together with exact *p*‐values and effect size measures.

## 3. Results

Based on the descriptive study and inferential statistical analyses, the data indicate that the Artemisia groups exhibited the highest significant antimicrobial activity, followed by white vinegar, while both treatments showed a significant reduction in CFU counts compared with the control group.

### 3.1. Comparison of Log Reduction Between Artemisia and White Vinegar (Heat‐Polymerized Group)

To evaluate the antibacterial efficacy of Artemisia and white vinegar against *S. aureus* within the heat‐polymerized acrylic group, log reduction was calculated for each substance. This value was determined by subtracting the mean microbial growth of the treated group from the mean value of the distilled water control group.

Statistical analysis revealed that Artemisia yielded a substantially greater microbial reduction (4.11 log cycles) than white vinegar (1.81 log cycles) in the heat‐polymerized resin specimens. Furthermore, the reduction was slightly greater in heat‐polymerized compared with CAD/CAM acrylic specimens, indicating a higher antimicrobial response under the tested conditions.

### 3.2. Comparison of Log Reduction Between Artemisia and White Vinegar (CAD‐CAM Group)

The antibacterial efficacy of Artemisia and white vinegar against *Staphylococcus aureus* was compared within the CAD‐CAM acrylic group by analyzing the log reduction produced by each treatment.

The results indicate that Artemisia outperformed white vinegar in the CAD‐CAM group, demonstrating a significantly greater antibacterial effect with a mean reduction of 3.42 log cycles, whereas white vinegar recorded a mean reduction of 1.20 log cycles. However, the magnitude of reduction was slightly lower than that observed in the heat‐polymerized acrylic specimens.

### 3.3. Study the Differences Between Treatments (Comparison of Heat‐Polymerized Groups)

The Kruskal–Wallis test was used to compare the effectiveness of the three treatments in the heat‐polymerized groups. The analysis revealed highly statistically significant differences among the treatments (*p* < 0.05). Based on mean rank values, Artemisia demonstrated the highest efficacy (lowest bacterial growth), followed by white vinegar, while distilled water showed the lowest effectiveness.

To identify pairwise differences between treatments, post hoc comparisons were performed using the Kruskal–Wallis test, which revealed significant differences among the three disinfection groups (*p* < 0.001). Dunn’s post hoc test with Bonferroni adjustment demonstrated significant differences between the tested groups (Table 2). The post hoc analysis confirmed statistically significant differences between all treatment pairs. Artemisia was significantly more effective than both white vinegar and distilled water, and white vinegar was significantly more effective than distilled water.

**Table 2:** Pairwise comparison of heat‐polymerized groups.

Samples	Test statistic	Std. error	Std. test statistic	Sig.	Adj. sig.^a^
*Artemisia*–white vinegar	−10.000	3.899	−2.565	0.010	0.031
*Artemisia*–distilled water	−20.000	3.899	−5.129	<0.001	0.000
White vinegar–distilled water	−10.000	3.899	−2.565	0.010	0.031

^a^Significance values have been adjusted by the Bonferroni correction for multiple tests.

### 3.4. Study the Differences Between Treatments (Comparison of CAD/CAM Groups)

The Kruskal–Wallis test was applied to evaluate the effect of the treatment materials (Artemisia, white vinegar, and distilled water) on CAD/CAM groups. Similar to the findings observed in the heat‐polymerized groups, the Kruskal–Wallis test revealed significant differences among the three disinfection groups (*p* < 0.001). Dunn’s post hoc test with Bonferroni adjustment demonstrated significant differences between the tested groups. Artemisia exhibited the highest effectiveness in inhibiting bacterial growth.

Pairwise comparisons revealed statistically significant differences in favor of Artemisia when compared with both white vinegar and distilled water, as well as in favor of white vinegar compared with distilled water in CAD/CAM samples (Table 3).

**Table 3:** Pairwise comparison of CAD/CAM groups.

Samples	Test statistic	Std. error	Std. test statistic	Sig.	Adj. sig.^a^
*Artemisia*–white vinegar	−10.000	3.900	−2.564	0.010	0.031
*Artemisia*–distilled water	−20.000	3.900	−5.129	<0.001	0.000
White vinegar–distilled water	−10.000	3.900	−2.564	0.010	0.031

^a^Significance values have been adjusted by the Bonferroni correction for multiple tests.

### 3.5. Study the Comparison Between Fabrication Methods (Heat‐Polymerized vs. CAD/CAM)

The effectiveness of bacterial growth inhibition between heat‐polymerized and CAD/CAM acrylic was compared for each treatment separately using the Mann–Whitney *U* test.

### 3.6. Study the Comparison of Artemisia Effectiveness Between Groups

The Mann–Whitney *U* test demonstrated a statistically significant difference (*p* < 0.001) in log reduction values achieved by Artemisia between the two fabrication methods. Artemisia exhibited slightly greater effectiveness, as indicated by higher log reduction, in heat‐polymerized compared to CAD/CAM samples.

### 3.7. Study the Comparison of White Vinegar Effectiveness Between Groups

The results also revealed a statistically significant difference (*p* < 0.001) in the effectiveness of white vinegar between the two types of samples. White vinegar achieved a higher log reduction in heat‐polymerized compared to CAD/CAM samples.

## 4. Discussion

The selection of *S. aureus* was a strategic decision to address a notable gap in existing literature; while the study is heavily saturated with studies on Candida, the bacterial contributors to denture biofilms are often overlooked. Additionally, although sodium hypochlorite is a conventional disinfectant, this study sought to investigate natural EOs as safer alternatives. This approach aims to mitigate the drawbacks of harsh chemical agents, such as the discoloration of prosthetic resins and the irritation of the oral mucosa.

Maintaining oral health in removable denture wearers requires effective disinfection protocols, as denture surfaces provide a favorable environment for microorganism adhesion, proliferation, and biofilm formation. *S. aureus* readily colonizes acrylic denture surfaces and has been associated with denture stomatitis and other oral infections, particularly among elderly and immunocompromised individuals [14]. Consequently, identifying safe and effective denture disinfectants remains clinically relevant.

Recent studies have increasingly focused on natural antimicrobial agents for oral health management due to their potential efficacy and reduced risk of adverse effects. These agents primarily act by inhibiting microbial growth or preventing biofilm formation, a key factor in bacteria [15, 16]. Indeed, the literature indicates that conventionally cured acrylic typically exhibits greater surface roughness than CAD/CAM‐milled blocks, thereby promoting higher microbial retention. Consequently, the superior surface smoothness characteristic of CAD/CAM prostheses may play a crucial role in minimizing biofilm accumulation [17, 18].

In this context, white vinegar has been shown to possess antibacterial activity against *S. aureus*, primarily through its low pH and its ability to disrupt bacterial cell walls and intracellular enzymatic systems [19, 20]. The potent antimicrobial activity of *Artemisia herba-alba* EO against *S. aureus* and other Gram‐positive pathogens is primarily attributed to its high concentration of terpenoids, including α‐ and β‐thujone, camphor, 1,8‐cineole, etc. These bioactive compounds likely operate via multitarget mechanisms that disrupt critical bacterial cellular functions [21, 22]. However, the specific contribution of these compounds to the antibacterial activity observed in the present study cannot be confirmed as the chemical composition of the extracted EO was not characterized.

Despite these promising findings, most previous studies have evaluated these agents in general biological settings or on nondental surfaces. Comparative data on their antibacterial efficacy on different types of denture acrylic, particularly under standardized in vitro conditions, remain limited. To our knowledge, no previous study has directly compared the antibacterial effectiveness of white vinegar and *Artemisia herba-alba* extract against *S. aureus* on both conventional and CAD/CAM acrylic resins.

This study demonstrated that *Artemisia herba-alba* extract exhibited significantly superior antibacterial activity against *S. aureus* compared with white vinegar and distilled water in both acrylic types. The extract achieved more than 99.99% bacterial reduction within 1 h of immersion under the tested in vitro conditions, consistent with previous reports of its potent antibacterial properties [21, 23]. This enhanced efficacy is likely attributable to the complex and synergistic action of multiple bioactive compounds present in the extract, which may contribute to multitarget antibacterial mechanisms.

White vinegar showed statistically significant antibacterial activity compared with the control group. However, its efficacy against *S. aureus* was lower than that of *Artemisia herba-alba*, likely due to its reliance on a single active mechanism mediated by acetic acid. This finding aligns with earlier studies suggesting that vinegar may be more effective against fungal organisms than Gram‐positive bacteria with thick peptidoglycan cell walls [11, 19]. Therefore, further investigations involving different oral microorganisms and biofilm models are required to better evaluate their clinical applicability.

Although statistically significant differences were observed between heat‐polymerized and CAD/CAM acrylic resins in response to both antimicrobials, heat‐polymerized acrylic specimens showed slightly higher log reductions compared with those of CAD/CAM specimens. However, these differences were minimal and of limited clinical relevance. The slightly higher effectiveness observed in heat‐polymerized acrylic specimens may be related to differences in surface roughness and porosity, which influence bacterial retention and antibacterial penetration [17, 24]. However, since these surface properties were not directly evaluated in the present study, this interpretation should be considered with caution and requires confirmation through future studies incorporating surface characterization analyses.

Nevertheless, the small magnitude of these differences suggests that both antimicrobial agents remain effective across the fabrication methods.

Importantly, the clinical relevance of Artemisia‐based antibacterial activity is supported by previous clinical trials demonstrating promising antimicrobial effects in denture‐related applications [25]. However, the clinical safety and long‐term applicability of *Artemisia herba-alba* require further evaluation through additional assessments, including cytotoxicity, color stability, surface changes, and mechanical property evaluations. This supports the translational potential of the current findings and highlights *Artemisia herba-alba* as a promising natural alternative for further investigation as a denture disinfectant. Nevertheless, the EO composition may vary according to geographic origin, harvesting season, environmental conditions, and extraction procedures. Moreover, the EO composition of *Artemisia herba-alba* may vary according to geographic origin, harvesting season, environmental conditions, and extraction procedures. Future studies incorporating GC‐MS analysis and phytochemical standardization are recommended to improve reproducibility and allow more accurate comparison among investigations.

A limitation of the present study is that the microbiological evaluation was limited to *Staphylococcus aureus* as a representative bacterial strain. Although *S. aureus* has been associated with denture biofilm formation and denture‐related infections, denture stomatitis is a multifactorial condition commonly involving *Candida albicans* and multispecies biofilms with bacterial–fungal interactions. Therefore, the antibacterial effects observed in this study should be interpreted within the context of the tested bacterial model and should not be generalized to all microorganisms associated with denture stomatitis. Future studies incorporating multispecies biofilm models, including both bacterial and fungal microorganisms, are recommended to provide a more clinically representative assessment of denture antibacterial efficacy.

In addition, bacterial identification in the present study was based on conventional phenotypic methods, including Gram staining, catalase testing, and mannitol salt agar confirmation. Although these methods supported the identification of *S. aureus*, future studies may benefit from incorporating advanced molecular or proteomic identification techniques, such as PCR or MALDI‐TOF, to provide further strain‐level confirmation.

Another limitation of this study is the absence of a clinically established positive control, such as sodium hypochlorite or chlorhexidine gluconate. The objective of the present study was not to establish equivalence with conventional disinfectants but rather to evaluate the antibacterial potential of the tested natural agents under standardized experimental conditions. However, the primary objective of the present study was to compare the antibacterial reduction achieved by the tested natural agents rather than to establish their superiority over conventional disinfectants. The antimicrobial effect was evaluated by comparing the microbial counts before and after immersion, allowing the determination of the reduction in the viable bacterial load caused by each tested solution. Since the antibacterial efficacy of conventional disinfectants such as sodium hypochlorite has already been extensively documented, they were not included as an experimental comparison in the current design. Nevertheless, future studies incorporating standard disinfectants as positive controls are recommended to allow direct clinical benchmarking of the tested agents.

Within the limitations of this study, we conclude that:

• Both *Artemisia herba-alba* extract and white vinegar demonstrated antibacterial activity against the tested *S. aureus* strain on heat‐polymerized and CAD/CAM acrylic resins under the conditions of this in vitro study.
• *Artemisia herba-alba* extract exhibited superior antibacterial efficacy compared with that of white vinegar.
• The antibacterial effectiveness of both *Artemisia herba-alba* extract and white vinegar was slightly higher on heat‐polymerized acrylic specimens compared with CAD/CAM acrylic specimens; however, this difference appears to be of limited clinical significance.
• The pH of the tested solutions was not evaluated in the current study, which may have contributed to the observed antimicrobial effects, particularly for acidic solutions such as white vinegar.
• Future investigations should include physicochemical characterization, including pH measurement, to better explain the mechanisms involved.

## Author Contributions

**Joul Kassis, Jalal Fandi, Alaa salloum**, **and Mariette Droubi**: data acquisition and interpretation, drafted, critically revised the manuscript, design. **Mariette Droubi, Joul Kassis**, **Samar Alsalameh, and Alaa salloum**: conceptualization and design, data interpretation, drafted, critically revised the manuscript.

## Funding

This study did not receive any specific funding.

## Disclosure

Joul Kassis had full access to all of the data in this study and Mariette Droubi takes complete responsibility for the integrity of the data and the accuracy of the data analysis.

## Ethics Statement

This study was performed in compliance with the World Medical Association Declaration of Helsinki on medical research ethics and approved by the biomedical research ethics committee under the Reference Number DN‐230426‐770.

## Conflicts of Interest

The authors declare no conflicts of interest.

## Supporting Information

Additional supporting information can be found online in the Supporting Information section.

## Supporting information


**Supporting Information 1** CRIS checklist: Completed checklist for reporting in vitro studies (CRIS) for the experimental design and reporting standard.

**Supporting Information 2** Raw data: Complete primary numerical dataset containing standardized colony forming unit (CFU/mL) counts for *Candida albicans*, and surface roughness values across all evaluated groups and time intervals.

## Data Availability

The authors confirm that the data supporting the findings of this study are available within the article and through contacting the corresponding author directly.
