# Supplementary material for: Antibacterial Efficacy of Artemisia Extract and White Vinegar as Disinfectants for Acrylic Prostheses Fabricated Using Conventional and CAD/CAM Techniques: An In Vitro Study
Source: Int J Dent. 2026 Aug 12;2026:6892571. doi: 10.1155/ijod/6892571 (PMC13463728; doi:10.1155/ijod/6892571)
Supplement: Supplementary file 1 [file IJOD-2026-6892571-s001.pdf]

CRIS Guidelines (Checklist for reporting *In vitro* studies)

Section/Topic	Checklist item	Reported on page no
**Title and abstract**	Identification as an in vitro/laboratory study in the title<br>Structured summary of trial design, methods, results, and conclusions	1,3
**Introduction**		
Background and Objectives	Scientific background and explanation of rationale<br>Specific objectives or hypotheses	4
**Methods**		
Interventions	The intervention for each group, including how and when they were actually administered, with sufficient detail to allow replication	5
Outcomes	Completely defined pre-specified primary and secondary outcome measures, including how and when they were assessed	10
Sample size	How sample size was determined	9
Randomisation: Sequence generation	Method used to generate the random allocation sequence	9,10
Allocation concealment mechanism	Mechanism used to implement the random allocation sequence, describing any steps taken to conceal the sequence until interventions were assigned	8
Implementation	Who generated the random allocation sequence	N/A
Blinding	If done, who was blinded after assignment to interventions and how	N/A
Statistical methods	Statistical methods used to compare groups for primary and secondary outcomes	
**Results**		
Numbers analyzed	For each group, number of 'items' included in each analysis and whether the analysis was by original assigned groups	11

Outcomes and	For each primary and secondary outcome, results for each group	11,12
**Discussion**		13
Limitations	Trial limitations, addressing sources of potential bias, imprecision	
Generalisability	Generalizability (external validity, applicability) of the trial findings	
Interpretation	Interpretation consistent with results, balancing benefits and harms	
**Other information**		
Protocol	Where the full protocol can be accessed, if available	15
Funding	Sources of funding and other support	
